# Can the SYNTAX score predict mortality in patients with cardiac arrest?

**DOI:** 10.1590/1806-9282.20240647

**Published:** 2024-09-02

**Authors:** Aykut Demirkıran, Cihan Aydın, Serhat Örün, Mustafa Kaplangöray

**Affiliations:** 1 Tekirdağ Namık Kemal University, Faculty of Medicine, Department of Cardiology – Tekirdağ, Turkey.; 2 Tekirdağ Namık Kemal University, Faculty of Medicine, Department of Emergency – Tekirdağ, Turkey.; 3 Bilecik Şeyh Edebali University, Faculty of Medicine, Department of Cardiology – Bilecik, Turkey.

**Keywords:** Sudden cardiac death, Survivors, Sudden cardiac arrest, Myocardial infarction

## Abstract

**OBJECTIVE:**

Sudden cardiac death or arrest describes an unexpected cardiac cause-related death or arrest that occurs rapidly out of the hospital or in the emergency room. This study aimed to reveal the relationship between coronary angiographic findings and cardiac death secondary to acute ST-elevation myocardial infarction.

**MATERIALS AND METHODS:**

Patients presenting with acute ST-elevation myocardial infarction complicated with cardiac arrest were included in the study. The severity of coronary artery disease, coronary chronic total occlusion, coronary collateral circulation, and blood flow in the infarct-related artery were recorded. Patients were divided into two groups, namely, deaths secondary to cardiac arrest and survivors of cardiac arrest.

**RESULTS:**

A total of 161 cardiac deaths and 42 survivors of cardiac arrest were included. The most frequent (46.3%) location of the culprit lesion was on the proximal left anterior descending artery. The left-dominant coronary circulation was 59.1%. There was a difference in the SYNTAX score (16.3±3.8 vs. 13.6±1.9; p=0.03) and the presence of chronic total occlusion (19.2 vs. 0%; p=0.02) between survivors and cardiac deaths. A high SYNTAX score (OR: 0.38, 95%CI: 0.27–0.53, p<0.01) was determined as an independent predictor of death secondary to cardiac arrest.

**CONCLUSION:**

The chronic total occlusion presence and SYNTAX score may predict death after cardiac arrest secondary to ST-elevation myocardial infarction.

## INTRODUCTION

Sudden cardiac death/sudden cardiac arrest refers to an unexpected death or arrest from a cardiovascular cause that occurs rapidly out of the hospital or in the emergency room^1^. The presumption based on epidemiologic studies is that the most common cardiac pathology underlying all sudden cardiac deaths is attributable to coronary heart disease^2^. Ventricular tachycardia and ventricular fibrillation are the most frequent in the first hours of an infarction^3^. Ventricular tachycardia or ventricular fibrillation accounts for the majority of episodes. However, bradyarrhythmia is responsible for some cases of sudden cardiac death/sudden cardiac arrest^4^. The largest experience with acute ST-elevation myocardial infarction (MI) comes from the GUSTO-1 trial of 40,895 patients who were treated with thrombolytic therapy^5^. The overall incidence of ventricular tachycardia or ventricular fibrillation was 10.2%. Approximately 80–85% of these arrhythmias occurred in the first 48 h. These data do not include patients with SCD who do not survive until hospitalization. It has been estimated that more than 50% of deaths due to acute myocardial infarcts occur out of the hospital, and most episodes occur within 1 h of symptom onset^6^.

The aim of this study was to identify the coronary anatomy of patients with acute ST-elevation MIs complicated by sudden cardiac death or sudden cardiac arrest. More specifically, we studied the differences between sudden cardiac death and survivors of sudden cardiac arrest.

## METHODS

Patients presented with acute anterior and inferior myocardial infarction between January 2013 and March 2024 were examined. Patients with out-of-hospital cardiac arrest or in-hospital cardiac arrest were included in the study. Coronary angiography and primary angioplasty were performed as treatment immediately after the patient arrived at the hospital. Right and left coronary angiograms were obtained in multiple projections. None of the patients received fibrinolytics. Two experienced cardiologists who were blinded to angiographic data interpreted all coronary angiographies recorded upon admission. The principal angiographic and clinical data were entered into a database. The reduction in luminal diameter was visually estimated to be due to graded coronary stenoses.

The lesion was considered the culprit if it was a fresh occlusion at angiography. Epicardial blood flow in the infarct-related artery was graded according to the thrombolysis in myocardial infarction (TIMI) group definitions^7^. The occlusion was classified as acute if angiography showed a thrombus at the location of the occlusion or if a guide wire was able to pass through the occlusion easily. If there was no acute occlusion, the lesion with the most severe reduction was assigned as the culprit if lesion localization corresponded with the location of ST segment elevations.

The coronary arteries were divided into proximal, mid, and distal segments. The severity of coronary artery disease was scored using the SYNTAX score I^8^. Coronary chronic total occlusion was defined as an occluded coronary artery with TIMI 0 flow for at least 3 months. The Rentrop-Cohen method was used to categorize coronary collateral circulation^9^.

Patients with known heart failure and coronary artery disease (coronary angioplasty and previous MI), left bundle branch block, Brugada syndrome, QT prolongation, congenital short QT syndrome, Wolff-Parkinson-White syndrome, familial polymorphic VT, and sudden unexplained death were not included in the study.

### Statistical analysis

Descriptive statistics for baseline parameters of continuous variables with a normal distribution were presented as mean±standard deviation. Qualitative variables were presented as numbers and percentages. The significance of differences in the means of continuous variables was evaluated using the Student's t-test. Categorical variables were compared using the chi-square test or Fisher's exact test as appropriate. Univariate logistic regression analysis was used to determine the independent predictor of mortality after cardiac arrest. The sensitivity and specificity of the SYNTAX score 1 to predict death were analyzed by receiver operating characteristics (ROC) analysis. A p-value below 0.05 was considered statistically significant. All tests were performed using the SPSS 22.0 (SPSS Inc., Chicago, IL) software version.

## RESULTS

A total of 302 sudden cardiac arrests were examined. After exclusion criteria, 203 arrests associated with ST-elevation MI were included in the study. A total of 161 deaths occurred within 1 h after the angiogram. Notably, 42 patients were included in the survivors group. Demographic data on deaths and survivors are summarized in Table 1. The time from symptom onset to collapse in patients with ventricular fibrillation and out-of-hospital cardiac arrest secondary to acute coronary syndrome is unknown. The median time between collapse and angiogram was 22.5±11.2 min after in-hospital cardiac arrest. Anterior localization was statistically more frequent in all cardiac arrests secondary to acute coronary events (68.5 vs. 31.5%; p=0.05).

**Table 1:** Baseline characteristics of the study patients.

Variables		Deaths secondary to cardiac arrest (n=161)	Survivors of cardiac arrest (n=42)	All cardiac arrests (n=203)	p-value
Age		71.5±11	73.5±8	71.9±10.5	0.27
Male		78 (48.4)	12 (28.6)	90 (44.3)	**0.021**
Smoking		90 (55.9)	20 (47.6)	110 (54.2)	0.603
Hypertension		29 (18)	7 (16.6)	36 (17.7)	0.940
Alcohol intake		87 (54.0)	19 (45.2)	106 (52.2)	0.459
Obesity		103 (64)	23 (56.1)	126 (62)	0.295
LVEF (%)		34.9±10.3	38.2±7.7	35.6±9.9	0.086
ST-segment elevation					
	Anterior	109 (67.7)	30 (71.4)	139 (68.5)	0.643
	Non-anterior	52 (32.3)	12 (28.6)	64 (31.5)	0.643
Complaints at first arrival					
	Chest pain	17 (10.6)	33 (78.6)	50 (24.6)	**<0.01**
	Cardiac arrest*	144 (89.4)	9 (21.4)	153 (75.4)	**<0.01**

LVEF: left ventricular ejection fraction.

* Patients with ventricular fibrillation out-of-hospital cardiac arrest secondary to acute coronary syndrome.

Bold indicates significant values (p<0.05).

Angiographic data are summarized in Table 2. The infarct-related artery in all cardiac arrests was LAD, RCA, or CX in 63.1, 29.6, and 7.4%, respectively. The most frequent (46.3%) location of the culprit lesion was in the proximal LAD, followed by the proximal RCA (25.1%). Considering coronary dominance in all cardiac arrest cases, 59.1% left-dominant, 21.2% right-dominant, and 19.7% co-dominants were detected. Flow in the infarct artery was absent or severely decreased in 74.9% of all cardiac arrests. Rentrop grade 2–3 collaterals to the infarct-related coronary artery were present in 21.2% of the patients. There were no differences between the two groups concerning the location of the culprit along the coronary arteries, the presence or absence of collaterals to the infarct artery, and coronary dominancy. Left ventricular ejection fraction (LVEF) was 34.9±10.3% in deaths and 38.2±7.7% in survivors (p=0.086).

**Table 2:** Coronary angiography results of the patients.

Variables		Deaths secondary to cardiac arrest (n=161)	Survivors of cardiac arrest (n=42)	All cardiac arrests (n=203)	p-value
Location of culprit lesion on LAD		103 (64)	25 (59.5)	128 (63.1)	0.877
Location of culprit lesion on RCA		46 (28.6)	14 (33.3)	60 (29.6)	0.877
Location of culprit lesion on CX		12 (7.5)	3 (7.1)	15 (7.4)	0.877
Coronary dominancy					
	Left	94 (58.4)	26 (61.9)	120 (59.1)	0.475
	Right	37 (23)	6 (14.3)	43 (21.2)	
	Codominancy	30 (18.6)	10 (23.8)	40 (19.7)	
Chronic total occlusion other than IRA		31 (19.2)	0	31 (15.2)	**0.033**
	LAD	24 (14.9)	0	24 (11.8)	
	RCA	7 (4.3)	0	7 (3.4)	
	CX	6 (3.7)	0	6 (2.9)	
SYNTAX score		16.7±3.5	12.1±1.7	15.8±3.7	**0.031**
TIMI-flow grade					
	0–1	125 (77.6)	27 (64.3)	152 (74.9)	**0.084**
	2–3	36 (22.4)	15 (35.7)	51 (25.1)	
Collaterals to IRA*					
	0–1	129 (80.1)	31 (73.8)	160 (78.8)	0.372
	2–3	32 (19.9)	11 (26.2)	43 (21.2)	

CX: circumflex artery; LAD: left anterior descending artery; IRA: infarct-related artery; RCA: right coronary artery; SYNTAX: SYNergy between PCI with TAXUS and Cardiac Surgery; TIMI: thrombolysis in myocardial infarction.

* Six patients had two chronic occlusions.

Bold indicates significant values (p<0.05).

**Figure 1:**
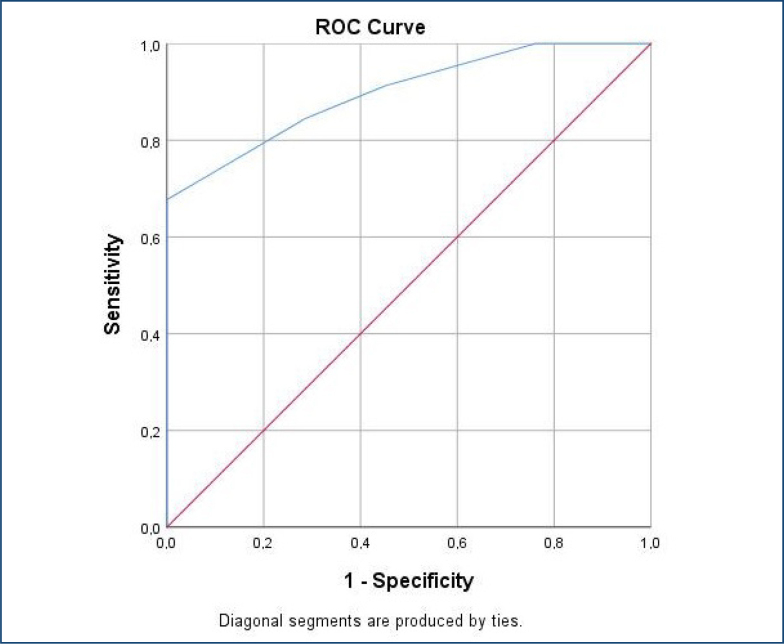
Determination of SYNTAX score cut-off value for predicting death.

Between survivors and deaths, there was a difference in the SYNTAX score and the presence of chronic total occlusion. Of all cardiac arrests, 31 (15.2%) had chronic occlusions in a non-infarct related coronary artery. All of the patients with chronic occlusion died within 1 h after coronary angiography, secondary to acute coronary events. On the contrary, in the survivor group, chronic total occlusion was not found (19.2 vs. 0%; p=0.02). It was found that the SYNTAX score was higher in the death group (16.3±3.8 vs. 13.6±1.9; p=0.03). The receiver operating characteristic (ROC) curve was used to test the accuracy of the SYNTAX score in the prediction of deaths secondary to acute coronary events. The optimal SYNTAX score cut-off value of 13.5 provided the highest sensitivity (84.5%) and specificity (71.4%) for predicting death. The area under the curve for the SYNTAX score was 0.898 (p<0.01) (Figure 1). A high SYNTAX score (OR: 0.38, 95%CI: 0.27–0.53, p<0.01) was determined as an independent predictor of mortality after cardiac arrest.

The mortality rate in patients with out-of-hospital VF was 89.4%. The mortality rate was found to be 10.6% in patients who presented with a complaint of chest pain (in-hospital cardiac arrest).

## DISCUSSION

According to our study results, the presence of chronic total occlusion and a higher SYNTAX score may be predictive of mortality after cardiac arrest secondary to ST-elevation MI. Additionally, the mortality rate in out-of-hospital VF was found to be higher than in-hospital cardiac arrest. Our finding is the increased risk of mortality with acute occlusion in the proximal LAD when compared with acute occlusion of the RCA or CX. The size of the infarct is correlated with early VF in large studies of patients hospitalized for AMI. Proximal LAD occlusion, which is associated with a large amount of myocardium, has a larger region at risk of necrosis^10^. However, it defies previous theories that acute RCA occlusions, which typically supply the conduction system, are more likely to result in potentially fatal arrhythmias^11^. Previous studies found associations between early VF and IRAs inconsistent, but some either included few or no patients with out-of-hospital VF or had no angiographic data. Other studies did not specifically address out-of-hospital VF secondary to ST-elevation MI and possibly included a heterogeneous group of patients with cardiac arrest^12^. In addition, a hypothesis, generated by previous studies, is that vagal tone in patients with acute occlusion of RCA protects against early VF during AMI. The cause of the vagotonia appears to be stimulation of cardiac vagal afferent receptors common in the inferoposterior of the left ventricle^13^.

One of our study findings is the presence of an association between mortality and the extent of coronary artery disease. The SYNTAX score was significantly higher in deaths secondary to acute coronary events. Our results conflict with autopsy data from patients who experienced an out-of-hospital cardiac arrest, where the degree of CAD was not substantially different from that of patients who had stable angina or a prior infarction^14^. This discrepancy could be explained by the different autopsy methods used to characterize the degree of CAD. According to Kyriakidis et al., there is a correlation between the Gensini score, which measures the extent of CAD, and in-hospital primary VF^15^.

Coronary dominance is defined based on the vascular supply of the posterior interventricular septum. When the interventricular septum is supplied by the posterior descending branch of the left circumflex artery, it is a left-dominant circulation. Left-dominant circulation is reported to be present in 2–10.1% of the general population^16^. However, we found the left dominance rate to be 59.1%. Several studies have attempted to determine the effect of coronary dominance on mortality as an outcome in patients with acute coronary syndrome. Observational data suggest that left-dominant circulation may be a risk factor for adverse outcomes^17^. Considering that our study population was sudden cardiac death or sudden cardiac arrest survivors, unlike the general population, and the left dominance was detected as more common, it can be thought that our study results support the previous studies.

In our study, mortality was found to be 89.4% in patients with cardiac arrest at the first admission to the hospital. Factors such as the time to reach the patient, the time to arrive at the hospital, and resuscitation experience determine mortality. Mortality among patients who collapse in an unmonitored setting correlates with the duration of the arrest^18^.

### Limitations

The study was somewhat limited by the small number of patients. Victims in whom resuscitation was unsuccessful may have had a different coronary anatomy from those who were successfully revived. Lack of knowledge about preexisting LVEF is one of the study limitations. These data are only accessible through prospective study designs, which are highly difficult to perform. Some risk factors that may be different between the two groups (sleep apnea syndrome, regular physical activity, depression, anxiety, psychological stress, and caffeine intake) were not recorded.

## CONCLUSION

According to our data obtained by acute angiography, LAD proximal lesions were the most common location in sudden cardiac arrest and sudden cardiac death secondary to ST-elevation MI. The presence of a chronic occlusion in a non-infarct-related artery or a higher SYNTAX score is possibly an additional independent determinant of mortality after a cardiac arrest.
